# Internal herniation through lesser omentum hiatus and gastrocolic ligament with malrotation: a case report

**DOI:** 10.1186/s13256-017-1471-4

**Published:** 2017-12-11

**Authors:** Ang Li, Renwang Hu, Dong Zhou, Senmao Li, Dan Huang, Xin Wei, Zhixin Cao

**Affiliations:** 1 Department of Gastrointestinal Surgery, Tongji Hospital, Tongji Medical College, Huazhong University of Science and Technology, No. 1095 JieFang Avenue, Wuhan, 430030 Hubei China; 2 Department of General Surgery, Xiangyang Central Hospital, No. 136 JingZhou Avenue, Xiangyang, 441021 Hubei China; 3 Department of Urology, Tongji Hospital, Tongji Medical College, Huazhong University of Science and Technology, No. 1095 JieFang Avenue, Wuhan, 430030 Hubei China

**Keywords:** Internal herniation, Lesser omentum hiatus, Malrotation, Case report

## Abstract

**Background:**

Internal herniation through lesser omentum hiatus and gastrocolic ligament with malrotation is extremely rare. This type of internal hernia has rarely been described before. Preoperative diagnosis is difficult and prone to misdiagnosis.

**Case presentation:**

A 38-year-old Chinese woman was an emergency admission to our hospital with a sudden onset of acute epigastralgia for the past 14 hours. We made a presumptive diagnosis of gastrointestinal perforation and septic shock. Due to the acute onset and rapid progress, she received timely surgical treatment. During operation, we observed that her small intestine herniated into the hepatogastric ligament and ligamentum gastrocolicum hiatus accompanied with intestinal malrotation that resulted in internal hernia. We found a diverticulum of approximately 3.0 × 6.0 cm sited at a distance of 80 cm from the ileocecal intestine. We resected the strangulated intestinal loop and the diverticulum, performed an appendicectomy, and closed the ligamentous fissure. Postoperation, she recovered smoothly, without any complications, and was discharged on day 6.

**Conclusions:**

A case of internal hernia formation is quite rare; accurate preoperative diagnosis and timely surgery are essential because it can cause strangulation of the ileus. However, the incidence of this internal herniation is low and preoperative diagnosis is difficult. An accurate preoperative diagnosis of internal hernia is still a challenge.

## Background

An internal hernia is formed in a variety of ways, such as various intestinal mesentery defects of congenital or acquired peritoneal folds. Duodenal hernia (53%) and pericecal hernia (13%) are the most common internal hernias, followed by the foramen of Winslow (8%), transmesenteric hernia (2%), and transomental hernia (1%) [1]. Internal herniation through lesser omentum hiatus and gastrocolic ligament with malrotation is rarely seen during a clinical examination. Preoperative diagnosis is difficult and prone to misdiagnosis. We report this case in order to enrich clinical thinking on acute abdomen.

## Case presentation

**Fig. 1:**
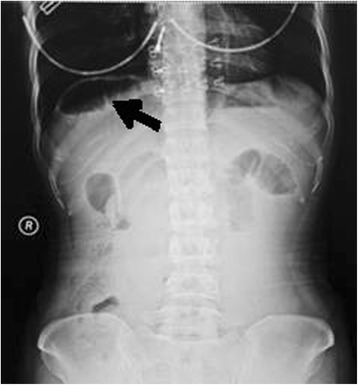
Abdominal X-ray plain film. The *black arrow* shows a gas loop of small intestine

**Fig. 2:**
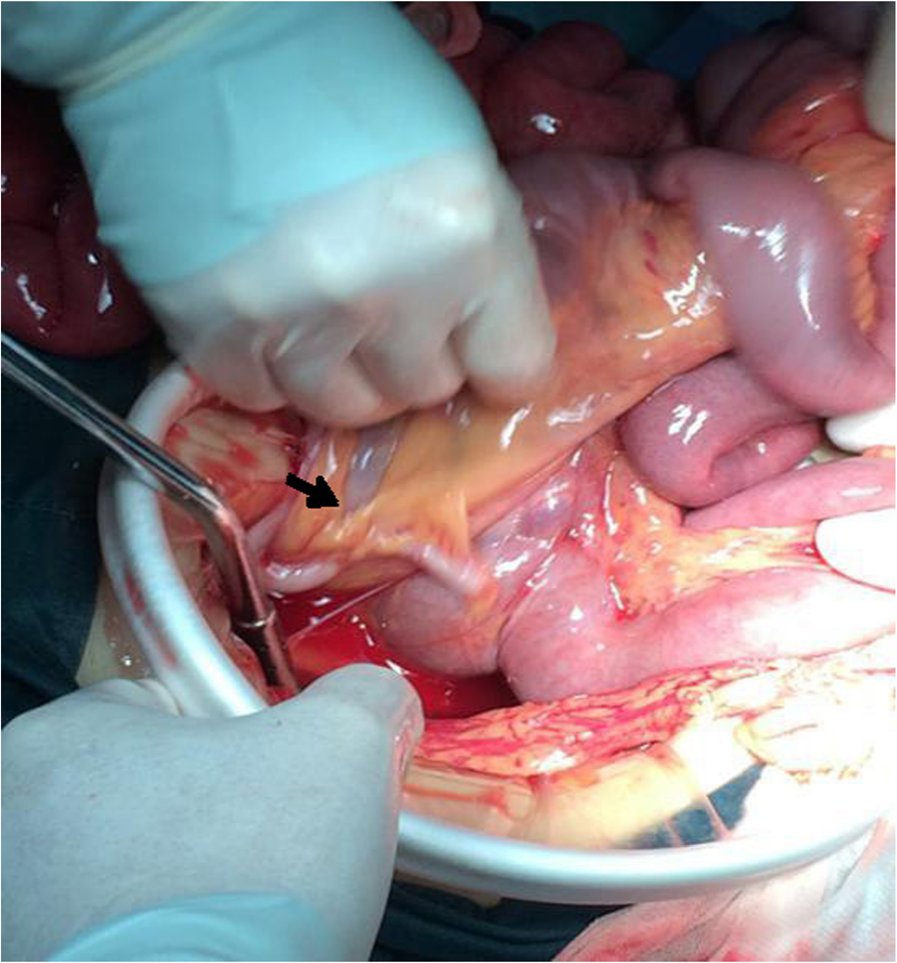
The ileocecal was free; the *black arrow* shows appendix

**Fig. 3:**
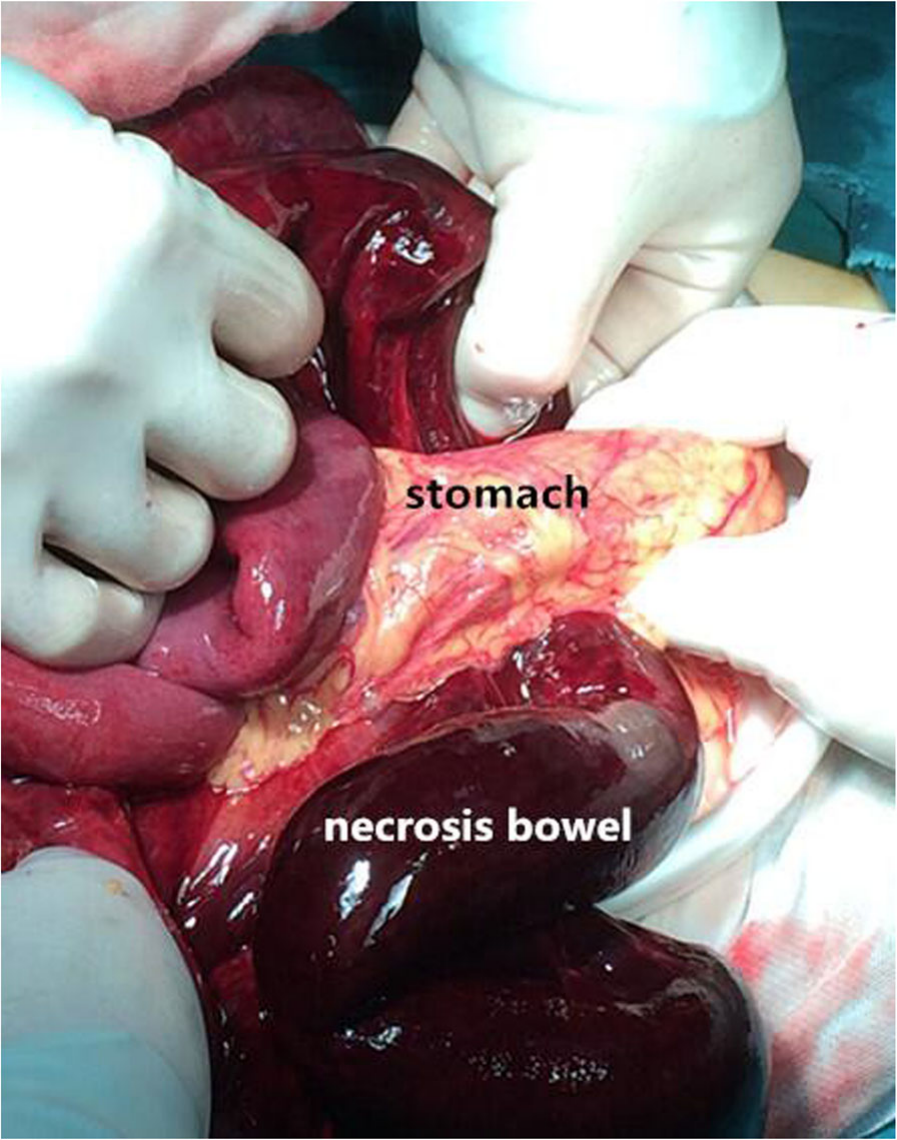
The small intestine herniated into retrogastric space. Intestine showing edema and necrosis. The *black arrow* shows stomach

**Fig. 4:**
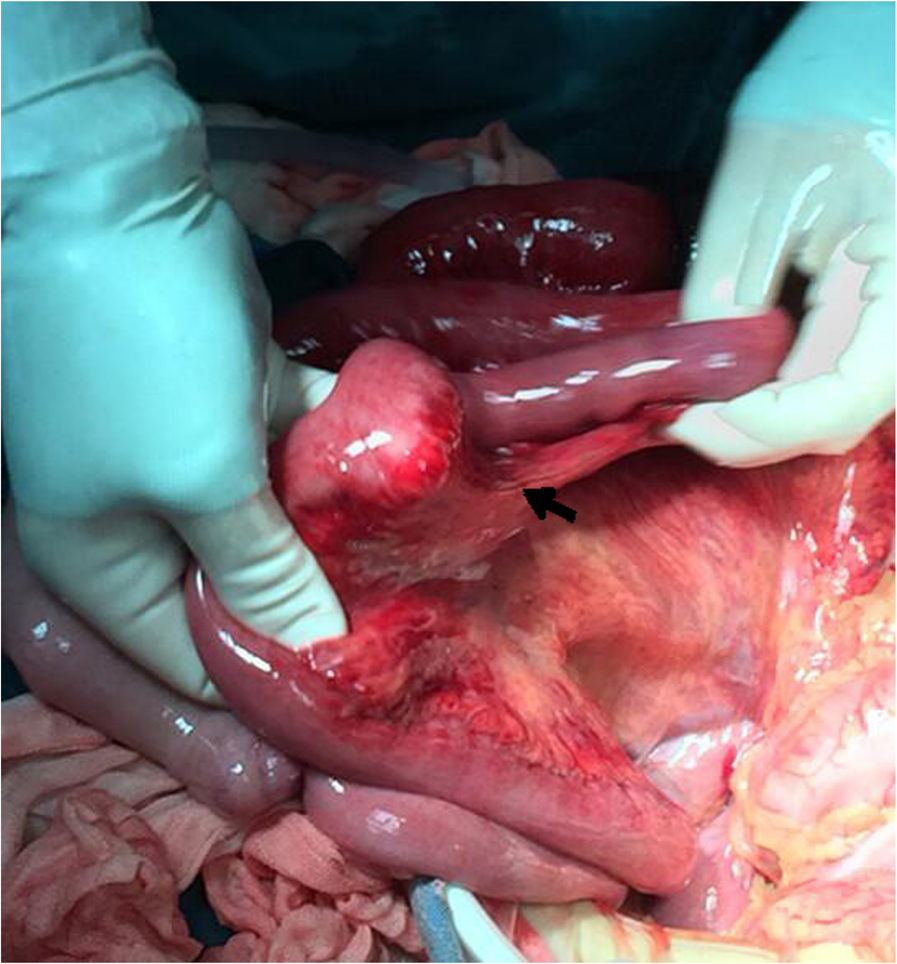
The black *arrow* shows diverticulum. Postoperative pathology suggested Meckel’s diverticulum

A 38-year-old Chinese woman was an emergency admission to our hospital with a sudden onset of acute epigastralgia for the past 14 hours. In particular, she had severe pain in her left upper quadrant, accompanied by nausea but no vomiting, inability to pass flatus and stool as well as intestinal obstruction, no radiating pain, and she was afebrile. She had a history of endometriosis but no abdominal surgery. On admission, she had pain and looked slightly pale, with a pulse of 102 beats/minute and a blood pressure of 106/77 mmHg. Her abdomen was tender in the epigastric region, but Blumberg’s sign and muscular defense were absent; bowel sounds were absent. Blood tests revealed an increased leukocyte count (15.41 × 10^9^/L, with 92% neutrophils), hemoglobin (Hb) 118 g/L, and normal liver and pancreatic enzymes. An emergency abdominal plain film (Fig. 1) demonstrated the right side below her diaphragm was free of gas; perforation of the digestive tract was considered. Her left upper jejunum had mild expansion, intestinal obstruction was not ruled out. After admission, she was dehydrated, hemodynamically unstable, and her blood pressure decreased progressively to septic shock. We made a presumptive diagnosis of gastrointestinal perforation and septic shock. Septic shock invariably requires anti-shock treatment and urgent surgery even if symptoms are limited as in our case. At laparotomy, we observed the ileocecal was relatively free (Fig. 2), which was pulled by the ileum at omental sac area, the small intestine loop dilated and herniated through the hepatogastric ligament hiatus into retrogastric space (Fig. 3), and then twisted out of a fissure in gastrocolic ligament. Approximately 1 m of her intestine showed edema; some of her small intestine was necrotic. Straightening some of her small intestine suggested intestinal malrotation for the cecum was free. There still was a diverticulum (Fig. 4) of approximately 3.0 × 6.0 cm sited at a distance of 80 cm from the ileocecal intestine. Finally, the strangulated intestinal loop and the diverticulum were resected, an appendicectomy was performed, and the fissure was closed. Her postoperative recovery was smooth and she was discharged on day 6 after the operation without any complications during the hospitalization.

## Discussion

Internal hernias, defined as the protrusion of a viscus through a normal or an abnormal aperture within the peritoneal cavity, are relatively uncommon clinical conditions, accounting for only up to 5.8% of small bowel obstructions, with an overall incidence of 0.5 to 0.9% [2], and lesser omental hernias are even rarer. Lesser omental hernias often occur in patients with a history of abdominal operation; an intra-abdominal hernia ring usually formed by an adhesion band accounts for approximately 63%. Compression of the intestine by the omental band results in volvulus or strangulated obstruction. Infrequently, congenital factors can cause internal hernia, such as long mesentery, intestinal malrotation, or intra-abdominal weak cracks or pores. In addition, there are congenital defects of the omental bursa, including hepatogastric ligament defects and an enlarged foramen of Winslow, which can lead to bowel obstruction when the small intestine herniates into these fissures and pores. For our patient, who had no history of abdominal surgery, an intraoperative exploration found that both hepatogastric ligament and gastrocolic ligament had a fissure accompanied with intestinal malrotation that strangulated internal hernia; this presentation has been rarely described. According to reports, midgut malrotation in adults is very rare, its incidence is approximately 0.2% [3].

Hernia of the omental bursa accounts for 1% of internal hernias [1]. In adults, omentum hernia sac is more commonly iatrogenic, mostly from Roux-en-Y anastomosis caused by gastric bypass surgery. The clinical manifestations vary with the condition of the herniated intestine; the manifestations are mostly acute onset and rapid progress. Abdominal pain, nausea, and vomiting usually emerge at an early stage and then symptoms of intestinal obstruction, a late manifestation of strangulating intestinal obstruction and infection, shock, and other systemic manifestations. In most instances, conservative treatment fails patients hospitalized with acute abdominal pain. The diagnosis is difficult because of its nonspecific symptoms and low incidence, so it is often diagnosed in emergency surgery. In this case, because the small omentum and ligament tissue structure were compact, the bowel hernia was prone to incarceration after herniating into these parts, ultimately leading to strangulation of the ileus and intestinal necrosis; our patient had diffuse abdominal pain, peritinonitis, serious infections, and circulatory disorders that caused septic shock.

Preoperative radiography could provide evidence for the diagnosis of a lesser omental hernia. Abdominal orthostatic X-ray is a valuable method, which shows a round or arc gas and air-fluid level in lesser omental bursa, anterolateral displacement of the stomach, and presence of intestine loops beneath the liver in the right upper abdomen. Unusually, however, this case showed an arc loop of small intestine in subphrenic, not in lesser omental bursa or beneath the liver; the imaging led to the misdiagnosis of a hollow viscera perforation at first sight! Moreover, the examination of multiple abdominal computed tomography (CT) scans plays an important role in the diagnosis of lesser omental hernia. Konishi *et al*. reported 16 cases of a small sac hernia and CT showed abnormal anatomic locations, including small bowel loops in the lesser sac or ventral to the stomach in 86% (6/7) of the patients, and convergence of the mesenteric vessels at the superior lesser curvature of the stomach in 43% (3/7) [4]. Another report described mesenteric vessels stretching anterior to the inferior vena cava and posterior to the portal vein [5]. However, there are no previous reports of a confirmed preoperative diagnosis [4]. Diagnostic abdominal puncture can support strangulated intestinal obstruction diagnosis as long as bloody fluid is detected. Clinically, there might be insufficient time to allow CT examination in patients with rapid progression of the disease; therefore, preoperative diagnosis is extremely difficult.

We speculate that the hernia occurred in this patient because an intestinal malrotation caused her internal anatomy to be abnormal, so that her intestine herniated into the vulnerable site of hepatogastric and gastrocolic ligament, then generated the internal hernia entity. Special attention should be paid to the diagnosis made before the operation. First, the abdominal plain film showed free gas below our patient’s diaphragm; from the first impression it was very easy to consider perforation of her abdominal cavity organs. Careful observation of the abdominal plain film reveals that under her diaphragm was gas loop of small intestine! The transverse arc subphrenic free gas is usually crescent shaped, while both ends of bowel loop shadow are often more rounded off (Fig. 1). Second, hollow viscera perforation peritoneal irritation is generally positive and our patient’s abdominal muscles were not tight, there was no obvious rebound pain, and her blood pressure was dropping and she had septic shock in a short time. These do not conform to the presentation of hollow viscera perforation. Of course, whether she had internal strangulated hernia or digestive tract perforation, if a patient has obvious symptoms and signs, then early operation is the treatment principle. Intraoperative diagnosis is surprising, but thanks to the timely treatment, our patient survived.

## Conclusions

The incidence of intra-abdominal hernia is rare, especially for patients who have not had abdominal surgery, so clinicians should be vigilant. Unfortunately, the preoperative diagnosis rate is low, patients often have obvious symptoms and late stage manifestation before emergency surgery. Because it is prone to incarceration and necrosis, delayed treatment could lead to dire consequences. Therefore, diagnosis and confirmation of preoperative diagnosis is still a challenge.
